# Experience of Physical Therapy Students Mentoring Older Adults with Health Literacy Tools

**DOI:** 10.1093/geroni/igab046.3013

**Published:** 2021-12-17

**Authors:** Mary Milidonis, Jane Keehan, Katherine Montgomery, Rebecca Deuley, Sara Formoso, Karen Kopera-Frye

**Affiliations:** 1 Cleveland State University, Hudson, Ohio, United States; 2 Cleveland State University, Cleveland, Ohio, United States; 3 Cleveland State University, Cleveland State University, Ohio, United States; 4 New Mexico State University, Las Cruces, New Mexico, United States

## Abstract

Health literacy is a top priority for Healthy People 2030. Healthy People 2030 defines personal health literacy as “the degree to which individuals have the ability to find, understand, and use information and services to inform health-related decisions and actions for themselves and others.” The purpose is to understand the experience of physical therapy students using health literacy tools with older adults to promote the adoption of health literacy tools in healthcare encounters. This project analyzes the reflection responses from students using qualitative methods. The qualitative methods included student reflection papers, word clouds, and focus groups. Twelve students participated in focus groups/ reflections. Thirty-seven students participated in word clouds. Health literacy tools included plain language, teaching teach back and “Ask me 3”®. Students were taught by student leaders and faculty about the meaning of health literacy and oral communication tools. Pairs of students provided health education with health literacy tools to older adults. Students then participated in a small group reflection to create word clouds. Students answered questions and provided five words that best answer each question. Students believed the benefits of health literacy tools for older adults includes better learning, participation and engagement. Reasons to use health literacy in the future were improved older adult independence, education and adherence. Students completed reflections and interviews at the end of the year to detail their experience with the health literacy tools. The pedagogical approach highlighted the value of experiential learning for the students while mentoring older adults.

